# Livestock Production in the UK in the 21^st^ Century: A Perfect Storm Averted?

**DOI:** 10.3390/ani3030574

**Published:** 2013-06-26

**Authors:** Christopher M. Wathes, Henry Buller, Heather Maggs, Madeleine L. Campbell

**Affiliations:** 1 The Royal Veterinary College, University of London, Hawkshead Lane, North Mymms, Hatfield, Hertfordshire, AL9 7TA, UK; E-Mails: heather@hcmconsulting.org.uk (H.M.); reprovet_uk@yahoo.com (M.L.C.); 2 Department of Geography, University of Exeter, Amory Building, Rennes Drive, Exeter, EX4 4RJ, UK; E-Mail: H.Buller@exeter.ac.uk

**Keywords:** livestock, sustainability, welfare

## Abstract

**Simple Summary:**

The global rise in demand for animal products for human consumption may well have an increasingly significant impact upon the natural environment, human health and the lives of farmed animals. This paper reviews some of the evidence for that impact and the future trajectories for livestock farming that it may well entail.

**Abstract:**

There is a school of thought that future demand for meat and other farm animal products is unsustainable for several reasons, including greenhouse gas emissions, especially from ruminants; standards of farm animal health and welfare, especially when farm animals are kept intensively; efficiency of conversion by livestock of solar energy into (human) food, particularly by pigs and poultry; water availability and usage for all types of agricultural production, including livestock; and human health and consumption of meat, eggs and milk. Demand for meat is forecast to rise as a result of global population growth and increasing affluence. These issues buttress an impending perfect storm of food shortages, scarce water and insufficient energy, which is likely to coincide with global population reaching about 9 billion people in 2030 (*pace* Beddington). This paper examines global demand for animal products, the narrative of ‘sustainable intensification’ and the implications of each for the future of farm animal welfare. In the UK, we suggest that, though non-ruminant farming may become unsustainable, ruminant agriculture will continue to prosper because cows, sheep and goats utilize grass and other herbage that cannot be consumed directly by humans, especially on land that is unsuitable for other purposes. However, the demand for meat and other livestock-based food is often for pork, eggs and chicken from grain-fed pigs and poultry. The consequences of such a perfect storm are beginning to be incorporated in long-term business planning by retailers and others. Nevertheless, marketing sustainable animal produce will require considerable innovation and flair in public and private policies if marketing messages are to be optimized and consumer behaviour modified.

## 1. Introduction

In 2009, Professor Sir John Beddington [1], when he was the UK Government’s Chief Scientific Adviser, predicted that “… by 2030 the world will need to produce 50 per cent more food and energy, together with 30 per cent more available fresh water, whilst mitigating and adapting to climate change. This threatens to create a ‘perfect storm’ of global events.” Anticipating such a storm and the need to weather it, we ask in this paper what are the implications for livestock farming, especially in the UK, and, crucially, for the lives of farm animals? We might also ask how accurate is Beddington’s forecast of a “perfect storm”? Might it be simply dismissed as a ‘worst case scenario’? Although some will contest the accuracy and validity of predicting that far into the future, planning for a range of eventualities can be valuable. Any attempt to look into a sustainable future of livestock farming should combine economic, technological, societal and environmental considerations. Some even question the very basis of livestock farming, often on ethical grounds.

Many scientists, politicians and others have attempted to define sustainable agriculture in the context of livestock production. In the United States, sustainable agriculture is defined legally (U.S. Code Title 7, Section 3103) as “*an integrated system of plant and animal production practices having a site-specific application that will over the long-term: satisfy human food and fibre needs; enhance environmental quality and the natural resource base upon which the agriculture economy depends, make the most efficient use of nonrenewable resources and on-farm resources and integrate, where appropriate, natural biological cycles and controls, sustain the economic viability of farm operations, and enhance the quality of life for farmers and society as a whole.*” Note that the American definition does not refer explicitly to animal welfare. We favour a definition that incorporates farm animal welfare, *i.e.*, a sustainable system of livestock farming is one that ensures the profitability of farming with minimal—or preferably no—impact on the natural environment, allows future generations to farm the land as they choose and allows farm animals to have at the, very minimum, a life worth living (from the animal’s point of view) with a growing number enjoying a good life [2].

Farm animals are kept for many purposes, most usually food, yet compared with plants they are relatively inefficient converters of solar energy into edible food for humans. While most people are omnivores, animal products nevertheless form an increasingly significant part of their diet. Global demand for meat, eggs, milk and many other livestock products is strong and growing, especially in consuming countries, such as India, with a large, growing population. The rapid growth of an affluent middle class in China, India and other developing countries implies a substantial and growing demand for livestock products, particularly food protein (these trends hold for other resources too). In Western countries, by contrast, changing meat preferences in particular have had a negative impact on certain types of meat production. Overall, from 2004–2006 to 2010, gross production of livestock per capita changed by −2.6% and +12.2% in the UK and China, respectively (see Table 1 also).

**Table 1:** Annual production of meat, eggs and milk in 2010 (source: FAO, [3]).

Country	Indigenous cattle meat (kT)	Hen eggs in shell (billions)	Cow milk, whole, fresh (kT)
**United Kingdom**	850	10	13,960
**China**	6,218	476	36,036
**World**	62,150	1,194	599,438

Any such growth would normally be associated with significantly increased agricultural production, assuming economic pressures of price elasticity and supply and demand. As Beddington and others anticipate, this means more intensive livestock systems, a growing proportion of arable land being given over to animal feed crops, greater competition over natural resources and an exacerbation of the environmental consequences associated particularly with such concentrated forms and spaces of husbandry [4,5,6].

Often called the ‘Nutrition Transition’, the emergence of middle classes with an increased demand for meat can result in the paradox of under- and over-nutrition in the same locale [7] and certainly at the global scale where the over-fed and the under-fed coexist in an unequal and increasingly competitive, some might say predatory, geography of access to nutrient availability. On the one hand, there are major and well documented concerns about the adverse impact of a diet rich in animal-based food on the health of those who eat much meat [8], as well as antibiotic resistance and livestock-derived zoonoses such as avian flu. Some commentators are concerned at the prospect of overly Western diets being taken up by millions of people in developing countries [9] with adverse consequences for human health and health economies. On the other hand, malnutrition, and particularly protein deficiency, continues to affect around 13% of the world’s population [10]. 

In the UK, the scale of use of farm animals has grown substantially over the past few decades, such that nearly a billion farm animals are reared annually, the majority of which are broiler chickens kept for meat. Britain is mostly self-sufficient in meat, eggs and milk with the notable exception of pig production; the number of sows kept for breeding has approximately halved since 1999. The sharp rise in the number of broiler chickens farmed is usually attributed to growth in demand, accompanied by technological advances, e.g., after the importation of hybrid strains from the USA since the Second World War. The benefit to us is a widespread availability of chicken meat.

Yet, accompanying this unequalled growth, in Britain and elsewhere, there are significant concerns about the economic rationale of global food consumption being so heavily dependent upon animal, rather than plant, products. Many criticize livestock farming because of the inherent inefficiency with which water, grass, grain and other feedstuffs are transformed into food. Consumption by livestock of soya beans is closely associated with loss of mostly South American rainforest as well as heightened emissions of carbon dioxide, methane and other green house gases, dust and ammonia, in addition to the growing problem of human and animal antibiotic resistance. The recent report to the United Nations by the Global Partnership on Nutrient Management [11] highlights the paradoxes of increased livestock production and consumption and the very real difficulties of achieving sufficient nutrients for an expanding global population if animal products continue to be an ever-growing source. For the World’s Society for the Preservation of Animals, the solution is clear: *“The need to slow and then reverse current growth in livestock numbers worldwide is inescapable, to protect both the environment and human health”* [12]. 

Finally, along with the more radical moral critiques of animal husbandry and meat consumption *per se*, there is fast growing ethical concern amongst consumers, amongst committed producers and amongst NGOs, particularly in the Western countries, about the quality of life of farm animals, particularly those animals kept in indoor intensive housing systems [13]. Quick to see the possibilities of market segmentation and brand fidelity offered by variable welfare standards, retailers and food chain actors in Western countries have responded by creating and developing new market opportunities [14] for animal products derived from production systems that exceed the requirements of regulatory minimum welfare standards [15].

Whether livestock should be used in intensive production systems at all is beyond the scope of this paper. However, it is widely accepted by many actors and commentators that highly adverse impacts on animal health and welfare might result from the intensification of husbandry in the name of ‘food security’ at whatever scale [16]. Although the ethics of different forms of animal husbandry—and for some, livestock farming as a practice in its entirety—have always been a topic of considerable debate, recent years have seen a growing and wider engagement, as demonstrated in 2011 [17]. Suffice it to say that people in most developed countries consume significant amounts of meat, eggs and milk produced by farm animals, which are nominally protected in law from extremes of suffering. However, there remain levels of suffering which are considered necessary and unavoidable for food production and are thus deemed lawful under British and many other countries’ legislation. What the definition and acceptable level of that ‘necessary suffering’ are is of course a critical area for social debate, ethical engagement, economic argument and scientific research.

Here then is a mix of potentially contradictory agendas for the future of livestock farming: ethics and economics, humans and animals, us and them, rich and poor, non-human welfare and human appetites, malnutrition and nutritious excess [18]. How might a way be charted through these opposing trajectories, how might the ‘perfect storm’ be avoided or, at the very least, mitigated?

One solution, which is fast adopting the status of global hegemonic discourse, is that of ‘sustainable intensification’. Another, perhaps less forcibly advanced, is that of ‘sustainable extensification’. In this paper, we wish to consider and confront these two approaches, looking in particular at their projected differential impact upon animal lives and the ‘Freedoms’ that have underlain animal welfare since first proposed by the UK’s Farm Animal Welfare Council in the late 1970s. We wish to argue that a notion of sustainable intensification that does not take into account the quality of animal lives is not sustainable at all. Arguments for sustainable intensification, for example, should address the animal welfare implications of the technologies and practices that this entails. Secondly, we maintain that concerns about food security and sustainability impact differentially upon different livestock systems. It has become commonplace to talk about ‘livestock’ and ‘livestock systems’ in a generic fashion [19]. However, certain types of livestock farming are, we maintain, possibly less able than others to weather Beddington’s storm even though global demand for animal products is certainly going to rise. Finally, we wish to re-emphasize that concern for human food security should not be at the expense of the very real gains in farm animal welfare that have been achieved over the last 40 or so years.

## 2. Sustainable Intensification

Faced with an inexorably rising global population and an increasingly aspirational global society for whom meat consumption seems to be uncritically equated with social, nutritional and cultural progression, the question of the sustainability of livestock production has been re-cast in terms of sustainable intensification, the new buzzword in global agricultural development, at least amongst the major Western actors in the global food community. 

Traditionally, sustainability has been defined as a balance of economic, environmental and social issues. However, farm animal welfare fits uneasily into this largely anthropocentric construction for which even the ‘environment’ has been largely misinterpreted in terms of maintaining accessible and finite natural resources for future generations of humans’ use. 

As we have argued elsewhere [20], farm animals are generally seen as, on the one hand, threats to sustainability (as either direct sources of pollution and environmental damage or indirect contributors through their impact upon land use, for example) [19] or, on the other hand, as vectors for the delivery of sustainability in high landscape or high biodiversity regions where low density grazing is seen as contributory to both environmental and socio-economic sustainability of rural areas [21]. In both scenarios, their value lies in their contribution—or threat to—human needs. Similarly, the welfare of farm animals is contrived principally as a critical part of economic sustainability, whether it is through concern for the productivity, and hence the welfare of farm animals as economic units or through concern for the social value of providing additional welfare as a sometimes costly public good. While some might also seek to place the welfare of farm animals as a ‘social’ concern, linked to ethics and societal recognition of farm animals as sentient beings, others still might argue that sustainability as a concept is too implicitly anthropocentric and that an animal’s quality of life should constitute a distinct fourth pillar within the sustainability edifice.

The recent UK Foresight Report [22] promotes *“sustainable intensification”* as the agricultural solution to the many perils awaiting humanity in a few decades time. Developed primarily as an objective for arable farming, with the increasing use of artificial (though ‘sustainable’) inputs and technologies, the phrase is increasingly used as a new paradigm for livestock farming in a world where the demand for animal products shows no sign of abating. Here though, the term becomes ambiguous [23], oxymoronic [24] or ‘dishonest’ [25], incapable of capturing the complex requirements when animals are farmed. Although there are many who argue that more intensive, well designed indoor husbandry systems can achieve welfare improvements through better and more efficient inputs, innovative forms of health and welfare monitoring, improved information, biosecurity, environmental management and waste disposal, such systems are also, inevitably, associated with an artificial, unnatural life that is not worth living, from the animal’s perspective [2] (Table 2). 

**Table 2:** Welfare issues of farm animals associated with intensive indoor housing systems (adapted from [26]).

Welfare Concerns	Welfare Advantages
Animals prevented from expressing natural behaviour and denied access to natural surfaces	New facilities built with specific welfare considerations
With large numbers, subtle indicators of an individual’s ill health or distress may be missed	Large facilities staffed by specialist veterinarians, nutritionists and stockmen to meet the specific needs of individuals and the herd
If a disease enters a herd or flock, it can spread rapidly	Protection from inclement weather. Reduces piglet mortality and nutritional stress for dairy cows
Reduced longevity of sows and cows due to the drive to increase production at the expense of fertility	Biosecurity can be enhanced by reducing exposure to, and from, wildlife

Moreover, in their reliance upon artificial inputs, such systems have a high risk of catastrophic welfare or health failure, capable of affecting a large proportion of a herd or flock. Finally, and even more likely perhaps in a market-driven environment, is a scenario where intensification is accompanied by de-regulation whereby animals are ‘engineered’ to high productivity and into exclusively all-year round indoor systems, consuming foodstuffs for rapid resource gain [27]: a veritable return to the ‘animal machines’ of Ruth Harrison [28] where under-performers, whether individuals or entire breeds, would be quickly removed if they did not conform to the system criteria. It is not surprising, therefore, that the concept of sustainable intensification of livestock production has led to concern that climate mitigation and food security *“will override ethical considerations and the goal of sustainable intensification will be used to justify systems of production that cause animal suffering”* [23].

Is this what we should consider as ‘sustainability’? There have been substantial gains over recent decades in farm animal welfare, at least in those predominantly Western nations in which food prices are relatively high and consumers are willing to pay for welfare standards above statutory minima. Such gains have not only improved farm animal lives, up to a point, but also have laid bare the excesses of the first major period of intensification that took place in the middle of the last century. In their pursuit of sustainable intensification, food chain actors and agricultural policy-makers should not repeat the mistakes of previous waves of intensification in agricultural and food policies, even though there appeared to be, initially at least, many apparent benefits of a policy of cheap food.

We believe that sustainable intensification should not be promoted at any cost. Our concern here is that the concept of sustainability must include the welfare of farm animals and must maintain the improvements that have been made to date. This should avoid the danger that sustainable intensification becomes the new hegemony, thus making it difficult for alternative systems (and arguments) to flourish. From our perspective, livestock agriculture simply cannot begin to approach the notion of sustainability if an animal’s life is not worth living.

## 3. Sustainable Extensification

An alternative trajectory for livestock farming in the face of Beddington’s ‘perfect storm’ is one that is less inclusive of all the major livestock sectors and less uniform in its geographical reach. Accepting that two of the components of the ‘perfect storm’ scenario are, on the one hand, the environmental, nutritional and trade implications of the ever-expanding animal feed industry and, on the other hand, the over-production/consumption of meat in particularly Western diets, any move towards a less intensive, pasture-based livestock sector, less dependent upon imported food and less in competition for land with more efficient forms of plant-based protein production might be seen as advantageous. Thus, responding, in part to the more positive contributions that livestock farming can make to the wider environment, proponents of sustainable extensification argue certain forms of livestock farming should be far more explicitly promoted in the hills and uplands of the UK, where grassland provides a ‘natural’ or ‘semi-natural’ feed in locations than where only ruminant animals can convert grass into food for humans and where other forms of intensive farming are unsuited. It might be argued that meat and milk from ruminants should be ‘encouraged’ in such areas, despite the general inefficiency with which solar-derived feed is transformed into edible food and other hazards of ruminant farming such as emissions of gaseous ammonia. Of course, the increased presence of grazing animals on formerly un-grazed pasture may also impact negatively upon biodiversity and a local reduction in those animal (and plant) species not so directly enrolled into the varying dynamics of human consumption.

In a recent report, the Institute for European Environmental Policy [21] explores three dimensions of the shifting livestock regime: reducing dependency on globally traded animal feed; increasing support payment mechanisms to encourage extensive pasture-based livestock regimes and promoting reduced consumption of low-value livestock products. With a reduction in feed imports, greater reliance would be placed on less-available home-grown feed, leading to a reduction in livestock production disproportionally affecting the intensive sector (pigs and poultry). Because ruminants can feed on a more diverse range of protein sources, including permanent and semi-permanent grasslands, than monogastric species which are generally fed on cereals, pulses and grains, which could be otherwise consumed directly by humans, areas dominated by such land cover would become more privileged sites of beef and sheep husbandry.

Finally, extensive forms of livestock production are often associated, rightly or wrongly, with higher perceived levels of animal welfare [29,30,31]. Yet care also needs to be taken with the unequivocal advocacy of production systems that necessarily equate outdoor access, however brief, as a universal welfare panacea and inherent component of sustainable livestock production. Although naming a husbandry system as ‘free-range’ may evoke many benign connotations of naturalness for consumers, numerous studies have recently demonstrated the sometimes significant welfare disadvantages that can result from poorly managed, free-range systems.

Within the overall context of free-market liberalism and de-regulatory government, consumer choice is likely to be a major driver in any such scenario. As the Institute for European Environmental Policy notes: *“If consumers can be persuaded to purchase meat and dairy products from more sustainable sources, for example through information, labelling and educational campaigns, this would further help to support extensive livestock producers”* [21].

## 4. Is Labeling the Answer?

Food production represents a complicated balance of interests. Western consumers have come to expect food that is plentiful, safe, of known provenance and cheap, although some commentators believe that the days of cheap food are coming to an end. The welfare standards of most farm assurance schemes go little further than compliance with statutory requirements. Nevertheless, they seek to confirm that all that legally ought to be done has been done, with independent auditing providing essential assurance to the regulator, retailer and consumer. Even then, as recent events have shown, control mechanisms fail, abuses occur and the messy complexities of integrating animal lives (and deaths) into human technological and economic rationales become apparent.

The concerned consumer frequently either lacks the information which s/he requires to make an informed choice at the point of sale or is overwhelmed by a confusion of labeling. Information provided about allergens, sell-by date, ingredients, calorific content and protein proportion and price is detailed and usually standardized. In all this, welfare labels are frequently misinterpreted when it comes to food miles, standards of animal welfare, livestock transport and other matters.

## 5. Conclusions

Given these concerns over livestock production, what are its prospects? We began by asserting that any attempt to look into the sustainable future of livestock farming should combine economic, technological, societal and ethical considerations, alongside any other policy or environmental concerns. Our concern is that these remain—as has been so often the case before—largely distinct and sometimes polarized considerations. On the one hand, population growth, affluence and global economic liberalism are driving what some have referred to as the neo-productivist paradigm in agriculture, a global rush to produce more meat, produce faster growing meat and with ever higher inputs through such approaches as ‘sustainable intensification’. On the other hand, another school of thought maintains that only by reducing the demand for meat can the global human population be fed sustainably in the future (*i.e.*, reducing the proportion of meat on the dinner plate as well as making meat and other livestock products more of a ‘one-off’ than a staple; [32]), with positive ‘knock-on’ effects for, at least, maintaining certain existing animal welfare standards. There is, of course, a range of other potential future solutions include using insects for food or *in vitro* meat production, or even reducing the global population, but these lie beyond the scope of this paper.

Garnett and Godfrey [23] advocate a more balanced approach to sustainability and intensification. However, behind this balancing, what is really needed is a more profound re-evaluation of needs and wants and a far greater research-led understanding of the linkages, interactions and impacts of these different trajectories, particularly with respect to the lives of individual animals. As we have argued elsewhere [33], to eat ethically, we should consume less and share more and, in the context of livestock animals, that also means acknowledging that our own lives are, as Dawkins [34] writes *“inseparably bound up with theirs”.* Born out of the dramatic expansion of intensive livestock farming in the middle of the last century, animal welfare science has, over the last 50 years revealed a great deal that was either ignored or unknown about the animals that provide us with food. If, as seems inevitable, at least in the medium term, global meat consumption is to rise then it must do so in the full recognition of the fact that the value of livestock cannot be limited solely to human nutritional and, in some cases, hedonistic functionality. In Beddington’s ‘perfect storm’, the danger is that the animal, as John Berger [35] once put it, again ’disappears’.
